# New-onset focal seizure as a presenting feature of HIV infection: a case report and mini review to the challenge in resource-limited settings

**DOI:** 10.1186/s12981-021-00344-0

**Published:** 2021-04-15

**Authors:** Biniyam A. Ayele, Zemichael Getu, Amen Samuel

**Affiliations:** 1 Department of Neurology, School of Medicine, College of Health Sciences, Addis Ababa University, Liberia Street, Lideta, 6396, Addis Ababa, Ethiopia; 2 School of Medicine College of Health Sciences, Addis Ababa University, Addis Ababa, Ethiopia; 3 Hayat Medical College, Addis Ababa, Ethiopia

**Keywords:** Cerebral toxoplasmosis, New-onset seizure, HIV, Sub-Saharan Africa

## Abstract

**Background:**

The frequency of new-onset HIV-associated seizure in the HIV-infected patient is estimated to be between 2 and 11%. Identifying the underlying etiology of new-onset seizure will have a vital impact on the mortality and morbidity of patients living with HIV infection.

**Case presentation:**

We report a 34-year old newly diagnosed HIV+ male patient presented with abnormal body movement (ABM) involving his right hemibody associated with loss of consciousness lasting few minutes of two weeks duration. The ABM occurred frequently (> five times per week) and associated with frothy and excessive salivation. He reported headache following each spells. Brain magnetic resonance imaging (MRI) showed bilateral frontal T2 and FLAIR hyperintensity and T1 hypointensity; post contrast study showed bilateral small ring enhancing lesion with perilesional oedema, the biggest one on the left hemisphere, with a 10 mm diameter; considering patient advanced immunosuppression and underlying HIV infection, the brain MRI findings were consistent with cerebral toxoplasmosis. Bipolar montage electroencephalography (EEG) study showed generalized background slowing, prominent in the left fronto-centeral region. Patient was managed with combination antiretroviral therapy, anti-toxoplasmosis medication, and anticonvulsant. On follow up, the frequency of seizure attack has significantly reduced.

**Conclusion:**

Considering the high prevalence of HIV infection and associated seizure among people living with HIV in sub-Saharan Africa, this case fairly highlights on the importance of utilizing advanced imaging techniques such as MRI and EEG in identifying the underlying causes of HIV-associated seizures.

## Background

Neurological manifestations occur in 50% to 80% of individuals infected with HIV virus and nearly 10% will have neurologic symptoms as the initial manifestation of HIV/AIDS [1, 2]. The frequency of new-onset HIV-associated seizure in the HIV-infected patient is estimated to be between 2 and 11% [2, 3]. Cryptococcal meningitis, tuberculoma, neoplasm, and HIV encephalopathy are the more common underling conditions associated with seizures in HIV-positive subjects [2, 4]. Identifying the underlying etiology of new-onset seizure will have a vital impact on the mortality and morbidity of patients living with HIV infection [4]. Determining the etiology and Neurolocalization of a new-onset seizure demands a detailed description of seizure semiology and through work up using brain magnetic resonance imaging (MRI) or CT scan and electroencephalography (EEG) [5]**.** In Ethiopia, the annual number of HIV infected people showed declining trends since 2002. Over the past two decades HIV prevalence rate decreased from 3.3% in 2000 to 0.9% in 2017 [6].

One of the major challenges related to HIV-associated seizure in resource limited settings such as sub-Saharan Africa (SSA) is universal lack of advanced neuroimaging and electrophysiological tests such as EEG [2]. Similar situation is evident in Ethiopia; nationally, only three public hospitals give an electroencephalography service. EEG service is universally absent in all urban, semi-urban, and rural cities except Addis Ababa and Mekele. To our knowledge, this is the first case report describing new-onset seizure as a presenting feature of HIV/AIDS from Ethiopia.

## Case presentation

We report a 34-year old right handed male patient from rural town in South Western Ethiopia, presented with abnormal body movement (ABM) involving his right hemibody associated with loss of consciousness lasting few minutes of two weeks duration. The abnormal body movement (ABM) occurred frequently (> five times per week) and associated with frothy and excessive salivation. He reported headache following each spells. In addition, he reported he had witnessed whitish oral lesions since the past two months. He was running a small private business. He has no history of fever, nuchal pain, motor weakness, and projectile vomiting. No history of illicit drug use, except khat chewing and occasionally drinks alcohol. No diabetes mellitus, hypertension, and cardiac illness. He was fully conscious and oriented. Neurological examinations were normal. Complete blood counts showed mild anemia and thrombocytopenia. His HIV serology test was positive. CD4 lymphocyte count was 185cells/uL. Anti-Toxoplasma gondii serology was not performed, because of patient’s financial problem.

**Fig. 1:**
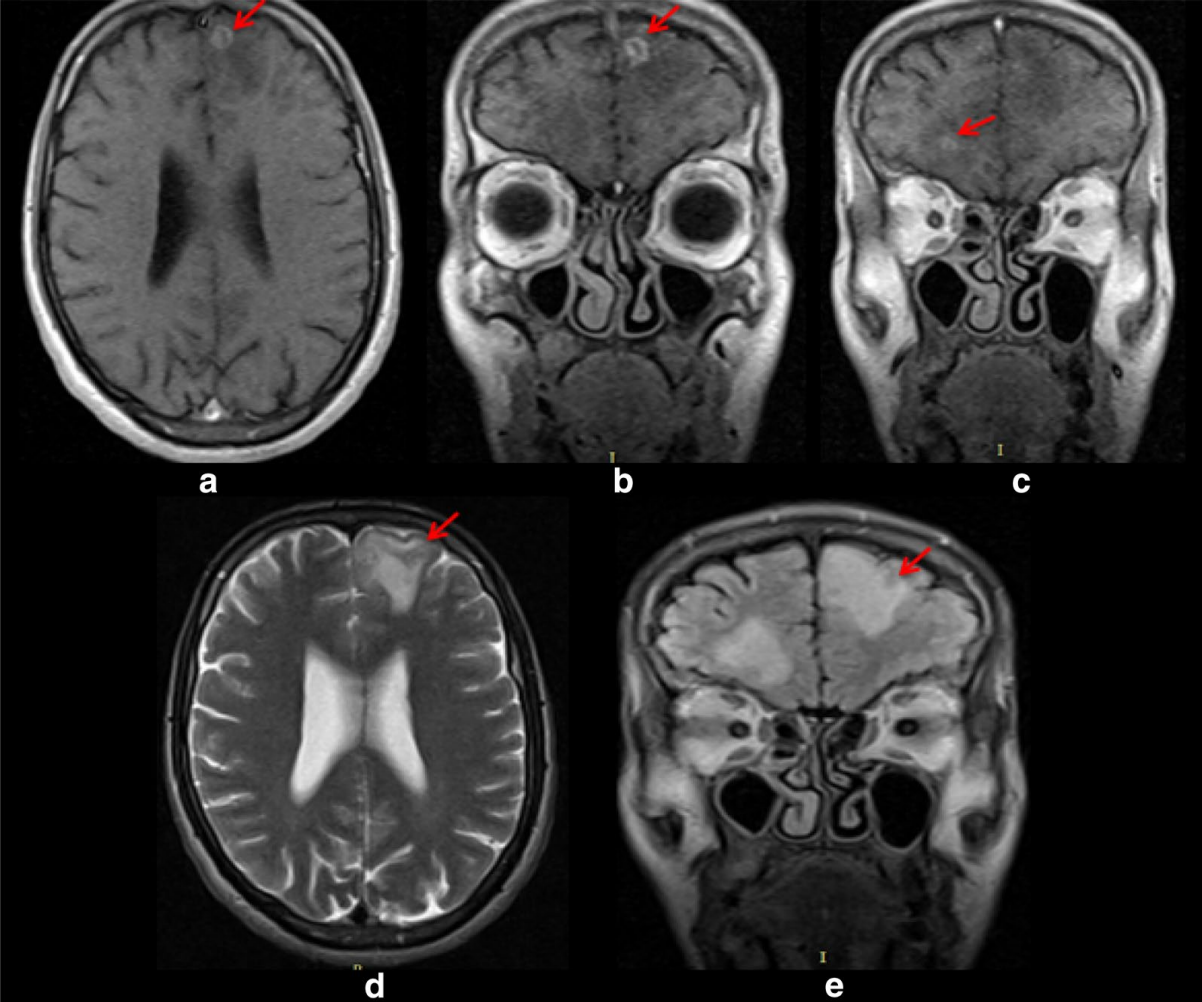
a–e: Post contrast T1 axial (**a**) and coronal brain MRI showing ring enhancing lesions in the left (**b**) and right (**c**) frontal lobal region. T2 axial (**d**) and coronal FLAIR (**e**) images showing perilesional edema (red arrows)

**Fig. 2:**
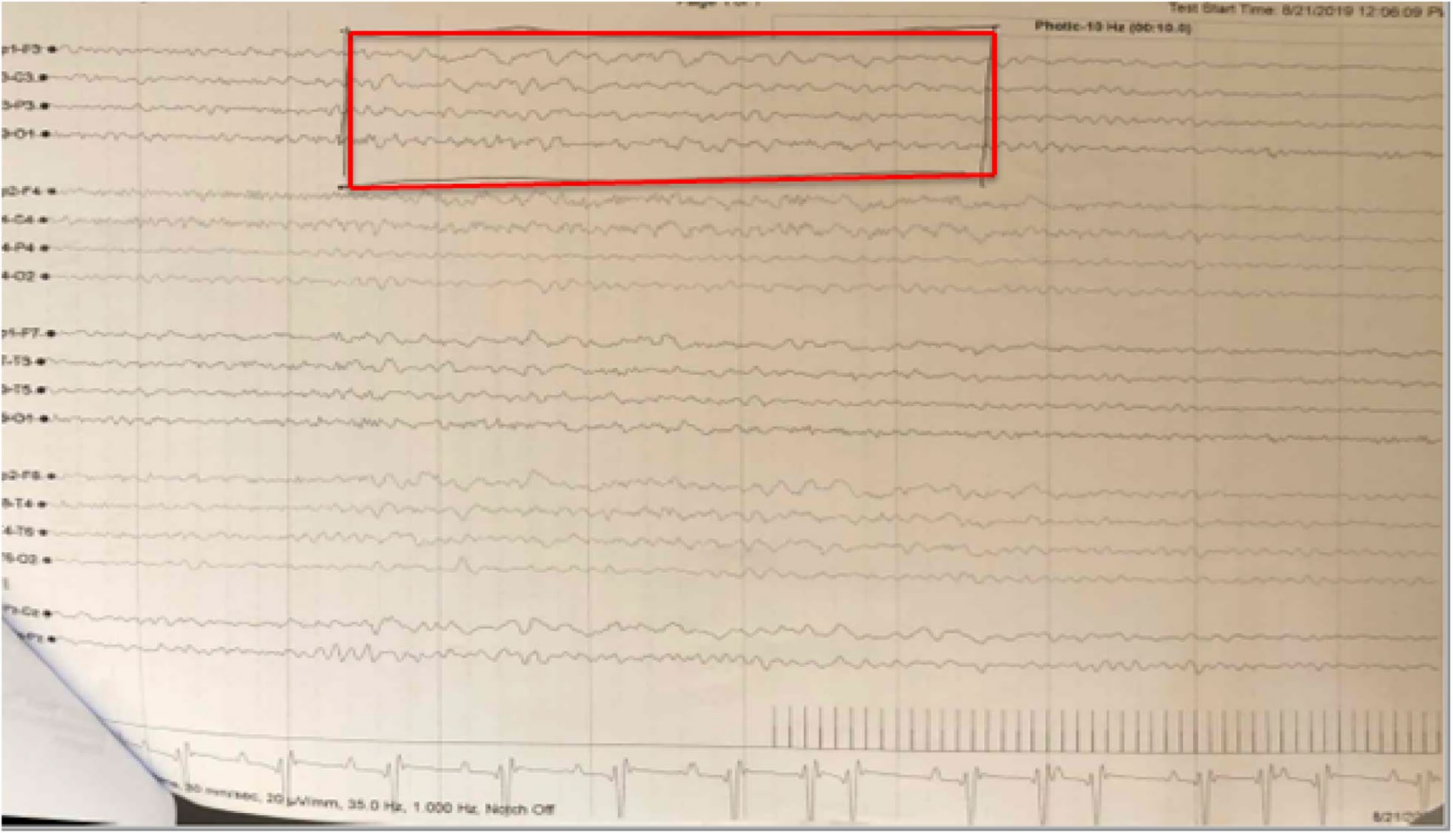
Bipolar montage electroencephalography study showed generalized background slowing, prominent in the left fronto-centeral region (red box)

He had 2–5 times elevated liver enzymes, normal kidney function, and Erythrocyte Sedimentation Rate (ESR) was 65 mm/hr. Brain MRI showed bilateral frontal T2 and FLAIR hyperintensity and T1 hypointensity; post contrast study showed bilateral small ring enhancing lesion with perilesional oedema, the biggest one on the left hemisphere, with a 10 mm diameter; considering patient advanced immunosuppression and underlying HIV infection, the brain MRI findings were consistent with cerebral (Fig. 1) toxoplasmosis. Bipolar montage electroencephalography study showed generalized background slowing, prominent in the left fronto-centeral region (Fig. 2). Subsequently, the patient was diagnosed with advanced HIV/AIDS presenting with focal motor seizure with impaired awareness likely secondary to cerebral toxoplasmosis infection, bicytopenia (mild anemia and thrombocytopenia), and elevated liver enzymes.

He was linked to chronic HIV care service and subsequently started on cART regimen containing Tenofovir/Lamivudine/Efavirenz. He is also started on anti-toxoplasmosis medication, Sulfamethoxazole 800 mg and trimethoprim 160 mg twice daily for 4 weeks and followed by 1 tablet daily for two months. In addition, the patient was started on sodium valproate 25 mg twice daily. Serum viral load and genotypic drug-resistance tests were not done as these services are not available at our setup. The patient was evaluated after fourth weeks of anti-toxoplasmosis treatment and one week after antiretroviral treatment. However, we couldn’t able to have a follow up brain imaging and monitoring of liver and renal function because of financial constrain. However, the frequency of seizure attack has significantly reduced.

## Discussion and conclusion

We report a newly diagnosed 34-year old HIV-infected male patient who presented with clinical semiology consistent with new-onset focal motor seizure with impaired awareness involving the right upper and lower limbs. Brain MRI showed symptomatic left frontal ring enhancing mass lesion and smaller lesion on left frontal region likely indicating CNS toxoplasmosis and EEG showed predominant left frontal focal slowing, further supporting the lesion on the left side was symptomatic. Considering his underlying HIV infection, he was started on cART, anti-toxoplasmosis medication, and anticonvulsant.

Toxoplasmic encephalitis (TE) is caused by the protozoan Toxoplasma gondii. Disease appears to occur almost exclusively because of reactivation of latent tissue cysts [7]. In patient with advanced HIV infection, CD4 cells below 200 are much vulnerable to develop TE [1, 3, 8]. Our patient was a newly diagnosed patient with advanced immunosuppression likely predisposing him to develop CNS toxoplasmosis. A study reported by Yacouba et al. 2014 [9] showed the overall prevalence of seizures among HIV-infected patient to be 45.2% and generalized seizure type to be the predominant type (75.8%) followed by partial seizure (15.2%) [9]. Our patient had a symptomatic focal lesion in the left frontal lobe, which likely explains the semiology of focal motor seizure on the right hemibody. Likewise, the associated loss of consciousness reported in the present case could be explained by the higher tendency of frontal lobe seizures to rapidly generalized to the contralateral hemisphere resulting in reduced mentation [10].

Wide variety of disorders may result in intracranial space occupying lesions (ICSOL) in individuals with HIV infection such as: tuberculoma, cryptococcoma, primary cerebral lymphoma, and bacterial brain abscesses [7, 9, 11, 12]. According to a review done by Pillay et al. 2018 [11] out of 110 brain CT scan of admitted HIV infected patients, 80.9% includes a differential comprising toxoplasmosis or tuberculoma [11]. This indicates TE is common among admitted HIV+ patients presenting with ICSOL. In resource-limited settings, one of the major questions about seizures and seizure disorders during the process of caring for people with HIV is to identify the underlying cause of the seizure [2]. Brain neuroimaging including MRI/ or CT scans and EEG are the two vital investigations helpful in identifying the underlying structural lesions which resulted in seizure disorder [7, 13]**.** Our patient had both brain MRI and EEG which showed a possible culprit lesion causing the symptomatic focal seizure. Early diagnosis, identification of underlying brain pathology, timely management of HIV-associated seizure is critical, because untreated and missed HIV-associated seizure is always associated with increased risk of mortality and morbidity among HIV infected individuals [1, 13].

## Identifying seizure causes—a major challenges in resource-limited settings

Identifying the cause of seizure is equally important to diagnosing seizure; because seizure recurrence is directly related to the presence or absence of underlying structural abnormality [2]. Furthermore, patients with advanced HIV infections are more likely to have symptomatic seizure as a result of causes such as: cerebral toxoplasmosis, tuberculoma, cryptococcoma, HIV-associated malignancies etc.[2, 3, 7, 9, 14]. In order to identify and classify seizure disorders, it’s important to have brain MRI/ or CT scan and EEG of the patients [13]. However, resource-limited settings such as sub-Saharan African region is universally known for its major neurological services and care deficiency [15–17]. Likewise, a 2004 report by WHO showed the number of specialists in neurology in Africa, at 0.03 per 100,000 population, is lower than in the other who regions [17]. To overcome such major challenge in resource limited settings, it’s advisable for care givers to depend on their clinical skills of Neurolocalization in order to determine if the patient had underlying structural abnormality. Our patient presented with new-onset right hemibody focal motor seizure associated with loss of consciousness. Therefore, we can clinically localize our patient’s symptoms to left hemispheric cortical region. Furthermore, the prominent motor features of our patient’s seizure semiology further help us to localize the lesion in frontal lobe [10]. Considering the high prevalence of HIV infection and associated seizure among people living with HIV in sub-Saharan Africa, this case fairly highlights on the importance of utilizing advanced imaging techniques such as MRI and EEG in identifying the underlying causes of HIV-associated seizures.
